# On Diseases of the Rectum

**Published:** 1838-04

**Authors:** 


### Art. XII.

*On Diseases of the Rectum.* By James Syme, f.r.s.e., Professor of Clinical Surgery in the University of Edinburgh.—*Edinburgh*, 1837. 8vo. pp. 138.

With the style and general arrangement of the present volume we are not inclined to find fault, nor with the greater part of the matter; but we entertain doubts whether such a work,—in which nothing actually novel is introduced, in which, in fact, there is only a concise reconsideration of the several diseases by which the rectum is affected,—was really required by the profession. If we had not read the preface, we might, indeed, have had some difficulty in determining whether the work were intended for the profession or for the public generally. Such passages as the following, for instance,—"A collection of matter is formed under the integuments of the hip;"—"but as the matter is situated between the skin of the hip and the mucous coat of the rectum;"—"the presence of a flat induration in the hip," and many similar which might be quoted,—seem so much wanting in precision that we could scarcely think them intended for medical men: and even the following sentence in the preface is rather ambiguous: "I have," says Mr. Syme, . . . . . "by a plain statement of the seat, nature, symptoms, and treatment of the different affections which are met with at the extremity of the rectum, endeavoured to assist practitioners in discharging their duty to the patient, and to protect patients against unprincipled or reckless practitioners." We would fain hope that our comprehension of this passage is correct, and that the author really means to protect the public by informing the practitioner; and yet, if this be his meaning, he is certainly chargeable with the want of precision. We cannot believe, however, that he has intended to do such a very silly thing as to seek to instruct those who cannot be substantially the wiser for his instruction; that he has attempted to make popular a subject which, above all others, is not likely to be mastered by the unlearned.

The first subject treated of is *Fistula in Ano*, respecting which the author says, "It is not easy to perceive why the slight incision required for its remedy is performed in the theatre of the hospital, with all the pomp and circumstance of a great operation." If it were a matter of moment, it would not be difficult to find excuses for this proceeding, although we believe the operation is very frequently done in the ward. Here, as in other cases, it is often desirable that the sufferers around should be as little as possible shocked by operations which are not necessarily done in the ward; and, besides, in the greater number of hospitals, there are students who are desirous of knowing something of such operations, and who have a better opportunity of acquiring this knowledge in a theatre than in the ward.—We think that, in enumerating the causes of abscess near the rectum, the author ought not to have passed over unnoticed hemorrhoidal tumours, which are perhaps one of the most frequent causes of such abscesses.

Mr. Syme states (p. 8,) "that, however long the fistula may be permitted to continue, no more than one internal opening is formed." Now, with the strongest moral conviction that this opinion is incorrect, our practice has never afforded us an opportunity of verifying, by an examination after death, the impression which existed on our mind. But, as the author thinks well enough of M. Ribes to quote him in support of his position, we will, from the same paper, quote him in support of ours: "In the greater number of cases which he has examined, (the whole amounting, in twenty-seven years, to eighty,) there was only one internal orifice; in some phthisical patients there were two; in one alone there were three." With regard to treatment, Mr. Syme says, "The inefficacy of all remedial measures except the knife for curing fistula, still remains unquestioned, unless by inaccurate observers or unprincipled empirics." This, we think, is stated too absolutely: there are, we believe, few experienced practitioners who have not had presented to their notice cases of fistula in ano, occurring in persons the state of whose pulmonary organs rendered it inexpedient to perform the usual operation, and yet in which it was necessary that something should be done for the present comfort of the patient. In such instances, stimulating injections have been used, and pressure prudently made; and now and then with complete success. We readily admit that this treatment is so uncertain, and the incision commonly so effectual, that no comparison between the two can hold: still this system has succeeded, and not only in such cases as we have indicated, and also in some cases in which no pulmonary complication was present, but in which the timidity of the patient, and perhaps also of the practitioner, has interfered to prevent the use of the knife.

It might be inferred, from the manner in which it is stated, that our author has been the first to discover that, in making the incision for the cure of this disease, it was unnecessary that the knife should be pushed to the end of the sinus. We know, however, that, for many years, the practice of our best London surgeons has been, when they have been unable to discover an internal opening, which is an unfrequent occurrence, to make the probe pass through the thinned portion of the rectum, and to incise the part intervening between this point and the sphincter. This point is never at the extremity of the sinus; neither is the internal orifice. The following passage, from the article *Fistula*, in the Dict. de Med. et Chir. prat., published several years back, establishes the same fact: "Formerly a great importance was attached, on the one hand, to incise the rectum as far as the superior limits of its denudation; on the other, to remove, with great accuracy, all the callous structure by which the fistula may be surrounded. Experience has done justice to the painful manœuvres to which practitioners had recourse to attain this double object. It is in the present day well demonstrated that, when all the parts comprised between the fistula and the intestinal canal are divided, and with them a certain extent of the sphincter ani, the portions of intestinal membrane which may remain floating and deprived of cellular tissue at the superior parts of the wound, are not long in adhering."

We object to the distinction which has been drawn in surgical works generally, as well as in those, like the present, especially devoted to diseases of the rectum, between hemorrhoidal tumours, according as they are external or internal. There is no sufficient reason for such distinction, and it does not appear to us to answer any good purpose: if their structure were absolutely dependent upon their situation, this arrangement would be unobjectionable; but it is not so. A varicose tumour may, and often does, exist at the margin of the anus; an erectile tumour is not unfrequently seen in the same situation; and another variety, which may be described as encysted, is commonly found there. We think it would be much better that the distinguishing character should be their structure alone. For instance: two tumours may be identical in structure; one may be situated a third of an inch superior to the other; one may, "distended by inflammatory engorgement, project beyond the sphincter," the other does not: of what use is such a distinction in this case?

The account given by our author of *Hemorrhoids* is far from complete, and is in several respects inaccurate. For the sake of our junior readers, we will here give a somewhat fuller view of these common and very distressing ailments.

1. Hemorrhoidal tumours, depending solely on the dilatation of a vein, often occur: they present a more or less elastic, irregular, lobulated swelling, of a bluish colour, and completely disappearing on pressure; they may bleed, either in consequence of an exhalation consequent upon distension, or of abrasion during the passage of indurated fæces; they may be complicated by the formation of a fibrinous coagulum within the cells, or by an effusion of lymph into the neighbouring parts, and then they do not disappear upon the application of pressure. They are now and then found almost surrounding the rectum, a little way above the anus, forming a kind of collar during the effort of stool; their development is gradual, and usually they are not very painful. These simple tumours, however, occur less frequently than the others.

2. A second variety of hemorrhoids have their seat in the dense submucous tissue of the rectum. They are roundish, more or less elongated, more or less tuberculated on their surface, sometimes pediculated, and of a palish red colour: their surface is sometimes bleeding, and occasionally dilated veins are seen to pass over them. When irritated and inflamed, they become very red and acutely painful: after having been inflamed they diminish, and as it were wither and become wrinkled, and may be seen pendent among the folds of the anus. These tumours sometimes disappear when the cause by which they were developed is no longer in action: under opposite circumstances, they increase and multiply. When recent, we find at their centre a cavity filled with blood; when of long standing, we may still find blood which has undergone change, surrounded by a delicate smooth cyst. At the internal surfaces of these cysts may be seen the orifices of very small vessels: these tumours are most probably a consequence of the extravasation of blood.

3. A third kind, which is commonly internal, is an erectile stricture; and we are quite at a loss to comprehend how Mr. Syme can have come to the conclusion that internal hemorrhoids can never have an erectile character, and "that erectile tissue must be congenital." Has he never known an erectile tumour in the uterus of a person advanced in life, or in the breast, or, in fact, in many other situations? and are not these accidental productions? Certainly this tissue is not an uncommon element in hemorrhoidal tumours; whether or not it be as frequent as a varicose state of the hemorrhoidal veins we are unable to determine. We are inclined to believe that, when hemorrhoidal tumours bleed periodically and freely, from very slight irritation, there is strong reason for suspecting that the hemorrhage does not proceed from the rupture of a varicose vein, but that it is the result of a vital action, of a transudation which occurs at the surface of an erectile tumour. When tumours of this sort are much distended and become ruptured, we perceive the spongy tissue of which they are formed, and not a single cavity dependent upon the dilatation of a vein. They are commonly single, varying in size from that of a pea to that of an egg. They frequently enter into a species of orgasm increasing in bulk, becoming very red, hot, and painful, and then exhaling arterial blood; and, when the irritation is past, returning to their former condition. The anatomical structure of these tumours seems to confirm the opinion of their nature: injection pushed into the hemorrhoidal arteries penetrates their substance, which then seems entirely vascular.

When inflammation attacks hemorrhoidal tumours its symptoms depend upon their character. In the case of the varicose tumour, there is burning heat, a sense of weight and distension; the varix soon opens, blood is poured into the rectum, and the symptoms speedily yield: sometimes the blood is poured out into the cellular tissue; lancinating, pulsating pain is felt; an encysted tumour is developed in the rectum or at the margin of the anus. When inflammation attacks an encysted tumour situated above the anus, there is lancinating pain with much heat, and a white puriform fluid, frequently mixed with blood, is discharged; after which the pain has suddenly ceased. If the tumour be projected beyond the anus, it becomes very red, hard, painful, and burning; the slightest contact augments the insupportable pain which the patient feels; afterwards it becomes softened, and, when opened, a puriform fluid, mixed with blood, escapes. In the treatment of "internal hemorrhoids" our author strongly advocates the employment of the ligature in preference to excision; and this, we think, is the proper as well as the prevailing mode of treatment: but, when he says, "that he feels warranted, after very extensive employment of the ligature, to state, that it may be used without the slightest risk of serious or alarming inconvenience," he states a circumstance which is opposed to general experience,—even "when the ligature is tightly drawn." To say nothing of the pain produced by it, numberless cases might be adduced in which peritonitis and phlebitis have succeeded to its employment: we hold, therefore, that it is never used without risk of serious or alarming inconvenience.

We can see no necessity for the change proposed by Mr. Syme in the signification of the term *prolapsus ani;* viz. to confine this term to those protrusions in which the whole thickness of the gut is concerned, the other forms of the disease being all referred to the head of "Hemorrhoids." If any change were necessary, we should say that it would be more reasonable to confine the term prolapsus to the descent of the mucous coat, and use the term invagination to distinguish the descent of the whole: this distinction is certainly founded on pathological anatomy. Of the cases which occur in practice, there is not one in twenty in which the muscular descends with the mucous coat; and, where it does, it is almost always a case of intussusception or invagination of the superior part of the rectum. Mr. Syme gives us nothing new on the subject of treatment.

We readily admit that great comfort may be afforded to the patient suffering from simple stricture of the rectum by bougies, but we do not, like the author, admit that they effect a cure, except in rare cases. Like a stricture of the urethra, it may be dependent upon induration of one or more of the tissues surrounding the canal, and may be dilated; but rarely will the tissue be softened or the dilatation become permanent. We do not quite agree with our author as to the best mode of using the bougie: instead of the immediate removal as soon as it shall have passed the obstruction, we would recommend that it should remain the greater part of an hour, if it can be borne without inconvenience by the patient. When once dilatation has been procured to a sufficient extent, it must be preserved by the occasional introduction of the bougie ever after.

Fissure of the anus should not be confounded with spasmodic stricture of the rectum. The author himself admits that he has met with fissures producing great pain to the patient, unaccompanied with spasmodic stricture, and with spasmodic stricture unaccompanied with fissure. "In a considerable proportion of cases," he says, "in which the sphincter is firmly contracted, there is no perceptible fissure."

The history of *Fissure* is not sufficiently well known, and we cannot say that our author has thrown any new light on it. What are its causes? A number are assigned; but we doubt if any of these serve to explain the suffering by which it is characterized. The anatomical lesion is apparently extremely slight, compared with the symptoms. It is believed that a fissure of the same apparent character above or below the sphincter is easily cured: is it, then, any connexion which is maintained with the muscular fibres of the sphincter that causes the suffering? Many cases are given in which the fissures occasioned violent pains, though they had no discoverable connexion with this muscle. Is, then, the spasmodic contraction cause or effect? It is certain that constriction is often met with without fissure, and fissure perhaps never without constriction: it is also certain that the section of the sphincter at any other point than that where the fissure is situated soon relieves the patient.

M. Boyer, for such reasons, maintained the first hypothesis; others adhere to the second, supporting their opinion upon the circumstance that ulceration may exist without being perceived. Although M. Boyer believed that the incision of the sphincter might be made anywhere, we think prudence would commend the choice of the fissured point: if, however, that point be at the median line anteriorly, the incision may be made at some other point of the circumference, as there may be some risk of wounding the vagina or urethra. Our author says it is not necessary to divide more than the external sphincter. We apprehend this is all which was desired by Boyer: still we should advise the surgeon to cut through all the muscular fibres which are called into action, or he may have to perform a second operation. The young surgeon must not, however, believe that the performance of this simple operation is without danger, and to be undertaken without careful deliberation; without, perhaps, attempting to cure the disease by dilatation, by lunar caustic, belladonna, and other means; as operations of this kind upon the pelvic organs not unfrequently occasion phlebitis.

We fear that certain cases of chronic inflammation of the rectum have been mistaken for cancer. The stricture, also, which attends an ulceration at this point occasionally misleads the practitioner, and is indeed a most fearful complication. It is, however, certain that, like a chronic ulcer of the stomach, which is now and then confounded with cancer of this organ, the chronic ulcer of the rectum occasions symptoms so analogous to those of cancer that it is confounded with it at the patient's bedside, and even sometimes upon the dead body. Stricture from chronic ulceration may be cured when it is accessible to dilating agents; and it is probable that it was in such cases, and not in true cancer, that Desault obtained such great success from gradual dilatation.

Our author says, "that, in common with other malignant affections, carcinomatous stricture of the rectum does not admit of being remedied by any kind of treatment, directed with the view of restoring the diseased part to its natural state; and its situation forbids any prospect of benefit from removal by the knife, or any other means." He further says, "If there are any cases in which this excision of the rectum has been followed by a permanent cure, the disease could not have been of a malignant nature." Now, we believe that carcinomatous matter, deposited spontaneously in a part in which chronic inflammation or ulceration does not precede it, is necessarily a disease of the system, and, so far as we at present know, irradicable; but we believe also that, where an irritation of a mucous, or perhaps cutaneous, surface has been long kept up, the part may take on a malignant character, and, unless removed, may end by destroying life; and yet the disease be purely local. Such cases, we apprehend, the author must have seen on the under lip, and on the penis: we would also maintain that a similar affection may exist in the rectum. In the lip, an ulcer may present all the characters of malignancy; but, if the neighbouring parts be unaffected, and the disease not too extensive, we may fairly hope for success: so in the penis; so, we believe, in the rectum. If the carcinoma of this part has succeeded to long-continued local irritation,—if it does not extend beyond the reach of the indicator finger; if the surrounding parts are healthy, the intestine moveable and susceptible of being brought down easily; if the disease have resisted ordinary treatment, and the patient's life be threatened,—we hold that excision should be attempted; and, unless this disease be very far advanced, we think it may be performed with a fair prospect of success. M. Lisfranc has now operated on a considerable number of cases, and with very considerable success. The parts removed by him have been examined by some of the most accomplished pathologists of the French capital, and there has been no hesitation in admitting that the structure was malignant—schirrous.

---

